# 

**Published:** 1861-08

**Authors:** 


					﻿Published Houthi), at $3 per Annum in Idiance.
K
k
S
k
c
5
•5
s
c
6
&
s
c*
2
g
i
c
k
I
I
k
■
>»
s’
i
Q
©
Cft
7
©
©
Z
z
k
s
<e>
aft
7
>0
x*
©
©
z
M
a
I
H
rjr
*
z
•c
ft.
. ATLANTA
9
JOURNAL.
J. G. WESTMORELAND, M. D.,
Pk.iEFSm.R Of M.TIKU MtDICA and MeI.ICAL JCBISPRl DESCK IS Tilt ATLAXT I M bleu (,'uLI.K.t,
EDITOR AND PROPRIETOR.
Pax er sclent la, sed verltas hint- tlniore.
y.rt- VI.	AUGUST, 1861.	Xo. XII
>B'rr.AJ^H^."l*H*iyaoKN< Pile vkin r.
Each Number contains Sixty-Four Pages.
*
o
*	I
=
K
J
ft
ft
9
X
ft
ft
1
ft
ft
£
*
0
ft
i
■«
*
$
>*
?*
s-
®
e
M*
K
8
* ■
5
0
ft
ft
5
©
ft
s
"i
«
ft
ft
7
ft
6
»
**
ft
ft
i
ft
ft
9
				

